# Repurposing Metformin to Promote Fracture Callus Maturation via AMPK‐Driven Metabolic Activation

**DOI:** 10.1002/jor.70246

**Published:** 2026-07-04

**Authors:** Vasyl Pastukh, Jianying Zhang, Peter G. Alexander, Satyaj Bhargava, Arshia Shams, Celina Zhao, MaCalus V. Hogan, James H‐C. Wang

**Affiliations:** ^1^ MechanoBiology Laboratory, Bethel Musculoskeletal Research Center, Department of Orthopaedic Surgery University of Pittsburgh School of Medicine Pittsburgh Pennsylvania USA; ^2^ Joint Tissue Development and Engineering Laboratory, Bethel Musculoskeletal Research Center, Department of Orthopaedic Surgery University of Pittsburgh School of Medicine Pittsburgh Pennsylvania USA; ^3^ Department of Bioengineering University of Pittsburgh Pittsburgh Pennsylvania USA; ^4^ Department of Physical Medicine and Rehabilitation University of Pittsburgh Pittsburgh Pennsylvania USA

**Keywords:** AMPK signaling, drug repurposing, endochondral ossification, fracture repair, mitochondrial biogenesis

## Abstract

Femoral shaft fractures cause prolonged disability, and therapies that accelerate bone repair remain limited. Repurposing clinically approved drugs that target biological bottlenecks in healing is a promising strategy. This study investigated whether systemic metformin administration, an anti‐diabetic medication with known metabolic regulatory effects, enhances fracture repair in a rat open femoral shaft fracture model. Histological, immunofluorescent, micro‐CT, and biomechanical analyses were performed at 6 weeks post‐injury comparing metformin‐treated and vehicle‐treated animals. Metformin markedly accelerated callus maturation, evidenced by earlier hyaline cartilage ossification, increased collagen I deposition and fiber organization, and reduced collagen II and III expression compared with controls. Micro‐CT analysis demonstrated increased tissue mineral density, trabecular thickness, and bone volume fraction along with reduced connectivity density, indicating more advanced structural consolidation of the callus. Although biomechanical parameters were not significantly different at intermediate time point, ultimate load and stiffness trended higher in metformin‐treated animals, consistent with structural advancement. Mechanistically, metformin increased p‐AMPK expression, elevated mitochondrial markers (NDUFB8, TFAM), and reduced extracellular HMGB1 release, suggesting enhanced metabolic capacity and attenuated inflammatory stress during repair. Importantly, metformin's effects were most pronounced during the cartilage‐to‐bone transition phase, supporting a role for metabolic activation in promoting endochondral ossification. Together, these findings demonstrate that systemic metformin administration promotes earlier structural consolidation of the fracture callus through coordinated metabolic and inflammatory modulation, supporting the potential repurposing of this safe and inexpensive drug as an adjunct strategy to enhance bone repair.

## 1 Introduction

Femoral shaft fractures are among the most serious orthopedic injuries, typically resulting from high‐energy trauma such as motor vehicle accidents, falls from height, or combat‐related blasts. They involve extensive soft‐tissue disruption and, in the case of open fractures, direct exposure of bone to the external environment, creating high risks of infection, delayed union, non‐union, and prolonged functional recovery [1]. Although these injuries occur most frequently in young adults, an increasing incidence in older adults is emerging with population aging [2, 3]. Despite advances in fixation and surgical care, therapeutic strategies that actively enhance intrinsic bone repair remain limited, motivating efforts to identify systemically deliverable interventions capable of improving biological healing outcomes.

Open femoral fractures are particularly dangerous due to severe hemorrhage, muscle and periosteal damage, and markedly elevated osteomyelitis risk [4, 5]. These injuries account for a disproportionate share of healthcare costs, disability, and lost productivity, and often require complex surgical management and prolonged rehabilitation [6]. Intramedullary nailing remains the gold‐standard fixation method, with plating or augmentation plating used selectively [7, 8, 9]. However, even with optimal fixation, severe trauma, infection, and systemic comorbidities may impair intrinsic bone healing and lead to delayed or incomplete repair [10, 11]. Therapeutic strategies capable of accelerating or augmenting bone regeneration—especially during the remodeling phase—are therefore urgently needed, particularly those that can be rapidly translated using clinically approved pharmacologic agents.

Bone healing progresses through overlapping inflammatory, reparative, and remodeling phases, each requiring coordinated interactions among immune cells, mesenchymal progenitors, vasculature, and mechanical stability [12]. Numerous biologics and cell‐based interventions, including bone morphogenetic protein −2 (BMP‐2), sclerostin inhibition, erythropoietin (EPO), mesenchymal stem cell therapy, and platelet‐rich plasma, have shown promise preclinically [13, 14, 15, 16, 17]. Yet their translation is limited by high cost, manufacturing complexity, inconsistent clinical benefit, and potential safety concerns. Safe, low‐cost, systemically deliverable therapies that can enhance callus remodeling remain highly desirable, creating strong interest in drug repurposing strategies that leverage existing safety and regulatory knowledge.

Metformin (Met), a widely prescribed oral anti‐diabetic agent, has recently emerged as a candidate metabolic regulator of skeletal repair. It has been shown to enhance angiogenesis, stimulate osteogenic differentiation through AMP‐activated protein kinase (AMPK)‐dependent autophagy and runt‐related transcription factor 2 (RUNX2) signaling, regulate stromal activity in diabetic bone, and promote osteoblastogenesis while suppressing osteoclast activity [18, 19, 20, 21, 22]. However, at least one report found no improvement in bone mass or fracture healing [23], suggesting that Met efficacy may depend on biological context—including inflammatory status, mitochondrial burden, dosing, and timing of delivery. These observations raise the possibility that repurposing Met may offer a practical strategy to enhance skeletal repair under conditions where metabolic and inflammatory stresses limit healing.

Mechanistically, AMPK activation is central to Met's cellular effects, regulating energy metabolism, osteoblast differentiation, autophagy, and skeletal homeostasis [24, 25]. Via the AMPK–PGC‐1α (peroxisome proliferator‐activated receptor gamma coactivator 1‐alpha) –TFAM (mitochondrial transcription factor A) axis, Met promotes mitochondrial biogenesis, a process critical for the high metabolic demand associated with endochondral ossification and late‐stage remodeling. At the same time, Met can suppress inflammatory signaling, including release of the damage‐associated molecular protein (DAMP) mediator high mobility group box 1 (HMGB1), which is strongly associated with delayed repair in traumatic bone injury. Open fractures often exhibit prolonged inflammation and insufficient metabolic capacity, making AMPK activation and HMGB1 suppression a theoretically synergistic therapeutic approach to enhance repair outcomes.

In this study, we tested the hypothesis that oral Met accelerates fracture healing by simultaneously enhancing AMPK‐driven mitochondrial activity and reducing HMGB1‐mediated inflammatory stress within the callus. Using a rat open femoral fracture model, we performed histological, immunohistochemical, micro‐computed tomography (micro‐CT), and biomechanical analyses to determine whether Met can advance the transition from cartilage to bone and promote earlier remodeling during fracture repair, thereby evaluating its potential as a systemically deliverable adjunct therapy to improve skeletal repair.

## 2 Methods

Female Sprague–Dawley (SD) rats (10 weeks old; total *n* = 38, Jackson Laboratories, Bar Harbor, ME, USA) were used for this study. A pilot experiment was first conducted using 3 rats/group at 3 post‐operative time points (4, 6, and 8 weeks), totaling 18 animals (9 vehicle and 9 metformin‐treated). A subsequent validation study focused on the 6‐week time point with 10 rats/group (*n* = 20). Female SD rats (10 weeks old) were selected as young adult, skeletally mature animals commonly used in fracture‐healing studies. Female rats also generally exhibit lower aggression and fewer fighting‐related complications during post‐operative housing than males. Animals were age‐matched and randomized between groups to minimize biological bias.

All animal procedures were approved by the University of Pittsburgh Institutional Animal Care and Use Committee (IACUC# IS00022685) and conducted in accordance with institutional guidelines, the National Research Council's *Guide for the Care and Use of Laboratory Animals*, and ARRIVE guidelines. Surgical anesthesia, post‐operative analgesia, and daily monitoring were implemented to minimize animal discomfort, and efforts were made to reduce animal use whenever possible.

An open mid‐shaft femoral fracture was surgically created in the right femur under 2% isoflurane inhalation anesthesia. Following aseptic preparation, a 1.5–2.0 cm lateral skin incision was made, and the musculature was bluntly separated to expose the mid‐diaphysis. The femur was transected using a 0.5‐mm oscillating saw blade, followed by stabilization using a retrograde intramedullary K‐wire inserted proximally through the greater trochanter and advanced across the fracture into the distal canal. The wound was irrigated, hemostasis achieved, and the fascia and skin were closed in layers. Post‐surgical antibiotics and analgesics were administered for 7 days to prevent infection and distress. Note that a standardized open osteotomy model was selected to provide reproducible fracture location, geometry, and fixation alignment across animals, thereby reducing inter‐animal variability in healing outcomes. Such controlled osteotomy models are commonly used in preclinical fracture‐repair studies evaluating biological interventions. Similar rat femoral osteotomy models using intramedullary fixation have been previously described and validated for fracture‐healing studies [26, 27].

After recovery, animals were randomized into two treatment groups. The Met group received daily oral gavage of 150 mg/kg Met (Metformin hydrochloride, Cat. #PHR1084, pharmaceutical secondary standard, Millipore Sigma, Burlington, MA) dissolved in 1 mL PBS, while control animals received an equal volume of PBS vehicle. The selected dose corresponds to commonly used rodent doses shown to activate AMPK without toxicity and approximates clinically relevant systemic exposure. Fracture‐healing outcomes were evaluated at 4, 6, and 8 weeks post‐injury. All rats were euthanized by carbon dioxide inhalation according to the approved institutional IACUC protocol. After confirmation of deep anesthesia or unconsciousness, thoracotomy was performed as secondary measures to ensure death.

### 2.1 Histological Assessment

Histological assessment for the main comparative analysis was performed at 6 weeks post‐fracture. Rat femurs were decalcified (*n* = 5 per group), longitudinally sectioned (6 μm thickness), and stained using Hematoxylin & Eosin (H&E), Safranin O & Fast Green (SO&FG), Masson's Trichrome (MT), and Picrosirius Red (PSR) techniques. Approximately 50 histological slides were prepared from each femur. Every 12th slide was selectively stained to assess tissue morphology, proteoglycan distribution, and collagen organization. All histological processing was performed by the University of Pittsburgh Biospecimen Core Facility following standard protocols. For semi‐quantitative histological analysis at 6 weeks post‐fracture, 4–5 animals per group were evaluated, with 2–3 sections analyzed per animal. Quantitative measurements from multiple sections were averaged to generate a single value per animal, and the animal was treated as the experimental unit for all statistical analyses.

### 2.2 Immunofluorescence Staining

Tissue sections from fractured femurs were used to evaluate the expression and localization of collagen types I, II, and III, as well as markers associated with metformin's mechanism of action, including AMPK, phosphorylated AMPK (p‐AMPK), NADH:ubiquinone oxidoreductase subunit B8 (NDUFB8), TFAM, and HMGB1. Each sample (*n* = 5 per group) was stained on four slides in‐house at the MechanoBiology Laboratory, University of Pittsburgh. Sections were incubated overnight at 4°C with primary antibodies against collagen type I (Abcam, ab260043), collagen type II (Abcam, ab307674), collagen type III (Abcam, ab7778), AMPKα1 (Invitrogen, MA5‐15815), p‐AMPK α1,2 (Invitrogen, PA5‐37821), NDUFB8 (Invitrogen, MA5‐37969), TFAM (Invitrogen, PA5‐29571), and HMGB1 (Invitrogen, MA5‐31967), all diluted 1:500 in blocking solution. After washing, sections were incubated with species‐appropriate fluorescent secondary antibodies (Invitrogen, A‐11001, A‐11008), counterstained with DAPI, and mounted with antifade medium. Immunofluorescence imaging was performed using a Keyence BZ‐X810 microscope.

### 2.3 Biomechanical Testing

A total of 16 rats (8 per group) were used for biomechanical evaluation at the 6‐week time point. A three‐point bending test was performed to assess mechanical strength at the fracture site. Specifically, each femur was positioned laterally on the testing apparatus, and load was applied from the medial to lateral side of the callus at a constant displacement rate of 0.1 mm/s until failure. Load–displacement curves were recorded, and deflection, ultimate load and stiffness were calculated. The contralateral (left) femur served as an uninjured control for normalization.

### 2.4 Micro‐CT Analysis

Micro‐CT was performed using a Scanco µCT 50 system (Scanco Medical, Brüttisellen, Switzerland). Scanning parameters were set to a voxel size of 10.3 µm, 55 kVp, 145 µA, 0.36° rotation step (180° angular range), and 1000 ms exposure per projection. Samples (*N* = 10/group) were scanned in 70% ethanol to prevent tissue shrinkage. Image reconstruction and analysis were performed using Scanco µCT software (OpenVMS; HP DECwindows Motif 1.6). Volumes of interest (VOIs) were manually drawn around the callus region, excluding the pre‐existing cortical bone and outer shell. Tissue segmentation used a global threshold of 0.5 g HA/cm^3^. The following parameters were quantified in the analysis: total volume (TV), bone volume (BV), and the bone volume fraction (BV/TV). Trabecular microarchitecture was evaluated by measuring trabecular number (Tb.N), trabecular thickness (Tb.Th), and trabecular separation (Tb.Sp). In addition, connectivity density (Conn.Den) and tissue mineral density (TMD) were also assessed.

### 2.5 Statistical Analysis

Data were analyzed using GraphPad Prism (Version 10.5.0, GraphPad Software, San Diego, CA). Normality was assessed using the Shapiro–Wilk test. Non‐parametric data (e.g., BV/TV data) were compared using the Mann–Whitney *U* test. Normally distributed micro‐CT variables and mechanical testing data were analyzed using one‐way ANOVA followed by Dunnett's post hoc test for comparisons against the control group. The intact femur group was included as a biological reference but was not used for treatment‐effect statistical comparisons. Also, the animal served as the experimental unit for all statistical analyses. Results are reported as mean ± SD, with *p* < 0.05 considered statistically significant.

## 3 Results

### 3.1 Qualitative Histological Analysis Indicates That Metformin Accelerates Fracture Callus Remodeling and Hyaline Cartilage Ossification

**Figure 1:**
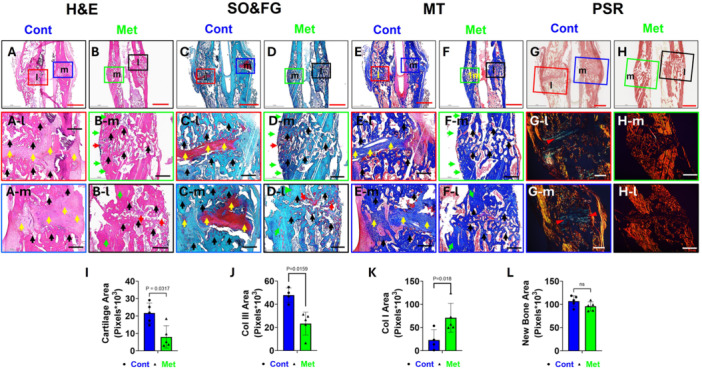
Metformin accelerates fracture‐callus remodeling at 6 weeks post‐fracture. Fracture calluses are presented as overview images (A–H) with enlarged lateral (l) and medial (m) regions of calluses. H&E‐stained sections show clear differences in healing progression between groups. In the control femur (A), the callus remains in an early remodeling stage dominated by hyaline cartilage (A‐l, A‐m, yellow arrows), with only peripheral zones of early woven/spongy bone formation (A‐l, A‐m, black arrows). In contrast, Met‐treated animals (B) exhibit a more advanced remodeling phenotype, with the fracture gap largely replaced by spongy/woven bone extending across the defect (B‐m, B‐l, black arrows). Early cortical bone remodeling is visible at periosteal and endosteal surfaces (B‐m, B‐l, green arrows), while only small residual cartilage zones persist centrally and peripherally (B‐m, B‐l, red arrows), indicating faster resolution of cartilage and accelerated endochondral ossification compared with controls. SO&FG staining further illustrates acceleration of the cartilage‐to‐bone transition. Control calluses (C) show intense proteoglycan‐rich cartilage staining (C‐l, C‐m, yellow arrows) with peripheral cartilage ossification initiating at the callus margins (C‐l, C‐m, black arrows). In contrast, Met‐treated calluses (D) contain predominantly mineralized woven bone throughout the defect zone (D‐m, D‐l, black arrows), along with ongoing periosteal and endosteal remodeling toward cortical bone (D‐m, D‐l, green arrows). Only small, faint proteoglycan‐positive areas remain, markedly fewer than in controls (D‐m, D‐l, red arrows), consistent with a later stage of endochondral conversion. MT staining demonstrates marked differences in matrix maturity. Control calluses (E) show patchy, incompletely organized collagen, and cartilage remains dominant within the defect space (E‐l, E‐m, yellow arrows), reflecting an early remodeling state, with woven bone formation only at peripheral regions (E‐l, E‐m, black arrows). In contrast, Met‐treated calluses (F) are characterized by dense and continuous collagen‐rich woven bone spanning intraosseous, periosteal, and endosteal surfaces (F‐m, F‐l, black arrows and green arrows, respectively), indicative of mid‐to‐late remodeling progression. The appearance of fibrous bone transitioning toward cortical structure further supports an advanced repair stage in Met‐treated animals. PSR overview images of control and Met‐treated groups are presented in bright field (G, H) and polarized light (G‐l, G‐m, H‐m, H‐l). In the control group, collagen III deposition appears as yellowish‐green particles and fibers and predominates over collagen I; collagen III deposition is disorganized or directed perpendicular to the femoral axis (G‐l, G‐m, red arrows). Whereas in the Met‐treated group, the fracture healing site predominantly shows collagen I, detected as orange‐red particles and fibers with compact organization (H‐m, H‐l). Red bars: 2 mm; black bars: 500 μm; white bars: 500 μm. Cartilage and collagen III areas were significantly decreased, and collagen I area was significantly increased in the Met‐treated group (I, J, K); however, newly formed bone area was not affected by Met treatment (L) based on histological slides semi‐quantification (*n* = 4–5, 2–3 slides/sample). ns: not significant.

To determine whether systemic Met administration enhances fracture repair, histological evaluation of calluses revealed clear differences between treatment groups (Figure 1A,B). In controls, the fracture site was composed predominantly of cartilaginous callus (Figure 1A–l, A–m, yellow arrows), with limited early ossification restricted to peripheral regions (Figure 1A–l, A–m, black arrows). In contrast, Met‐treated rats exhibited extensive spongy/woven bone formation (Figure 1B‐m, B‐l, black arrows) and an ongoing transition toward cortical bone (Figure 1B‐m, B‐l, green arrows). Only small central islands of cartilage remained (Figure 1B‐m, B‐l, red arrows), indicating faster cartilage‐to‐bone replacement compared with controls. SO&FG staining corroborated these findings (Figure 1C,D). Control calluses showed intense proteoglycan content, reflecting persistent hyaline cartilage, along with patchy peripheral ossification (Figure 1C‐l, C‐m, yellow and black arrows, respectively). In contrast, Met‐treated calluses consisted primarily of bone rather than cartilage (Figure 1D‐m, D‐l, black arrows), with clear evidence of cortical remodeling (Figure 1D‐m, D‐l, green arrows). Only minimal residual proteoglycan staining remained (Figure 1D‐m, D‐l, red arrows), consistent with nearly complete endochondral ossification.

Additional temporal histological evaluation (Figure S1) demonstrated that at 4 weeks both groups remained in an early reparative/remodeling stage with prominent cartilage and immature callus tissue. The most distinct between‐group differences were observed at 6 weeks, when metformin‐treated animals exhibited more advanced cartilage replacement and callus maturation. By 8 weeks, both groups demonstrated progressed ossification and remodeling, with reduced intergroup differences.

Consistent with this mid‐phase–specific effect, histological comparison across time points revealed minimal differences between groups at 4 and 8 weeks, whereas the most pronounced acceleration of cartilage ossification occurred at 6 weeks (see Figure S1).

### 3.2 Qualitative Matrix Analysis Suggests Improved Collagen Deposition and Fiber Organization With Metformin

Masson's trichrome (MT) staining demonstrated greater collagen content and more advanced matrix maturation in Met‐treated fractures compared to controls (Figure 1E,F). Controls displayed irregular collagen distribution with incomplete bridging between fragments (Figure 1E‐l, E‐m, yellow arrows). Met treatment resulted in a dense, well‐organized collagen network spanning intraosseous, periosteal, and endosteal regions (Figure 1F‐m, F‐l, black arrows), accompanied by greater periosteal and endosteal collagen accumulation and minimal residual cartilage (Figure 1F‐m, F‐l, black arrows and green arrows, respectively). Picrosirius red (PSR) under polarized light showed corresponding collagen fiber differences (Figure 1G, H). Control calluses contained heterogeneous, poorly aligned fibers (Figure 1G‐l, G‐m, red arrows). Met‐treated fractures showed predominantly red–orange birefringence, indicating mature, tightly packed, axially aligned collagen fibers (Figure 1H‐m, H‐l).

The Met‐treated group showed a significant decrease in cartilage area (7.9 ± 6.3 × 10^3^ pixels) compared to control (21.6 ± 5.8 × 10^3^ pixels) (Figure 1I). A significant decrease was also observed in collagen III deposition area in the Met‐treated group (23.2 ± 9.9 × 10^3^ pixels) compared to untreated control (47.7 ± 6.3 × 10^3^ pixels) (Figure 1J). In contrast, collagen I area was significantly increased in the Met‐treated group (70.9 ± 31.2 × 10^3^ pixels) compared to control group (22.9 ± 22.3 × 10^3^ pixels) (Figure 1K). However, newly formed bone tissue formation area was similar in both groups; 95.7 ± 9.7 × 10^3^ pixels in the Met‐treated group, compared to 106.9 ± 11.6 × 10^3^ pixels in control group (Figure 1L). Full‐sized rat femur bones histological slides are presented in Figure S2.

### 3.3 Qualitative Immunofluorescence Analysis Shows Increased Collagen I and Reduced Collagen II/III With Metformin

**Figure 2:**
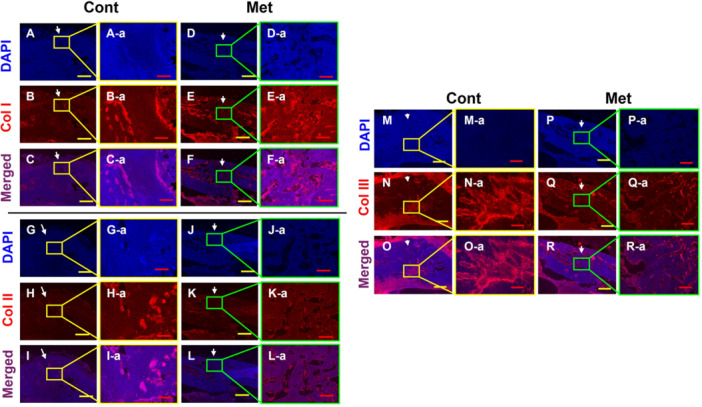
Metformin markedly increases collagen I deposition, suppresses collagen II, and reduces collagen III in the fracture gap. Immunofluorescence imaging reveals minimal collagen I within the defect space of control fractures (A–C), leaving a wide gap between fragments and limited bone matrix deposition (A‐a–C‐a). In contrast, Met‐treated femurs (D–F) exhibit robust collagen I expression spanning the fracture zone, indicating active woven bone formation and structural bridging (D‐a–F‐a). The white arrows denote fracture orientation across panels. In control calluses (G–I), collagen II remains abundant, consistent with persistent cartilage occupying the defect (G‐a–I‐a). In Met‐treated tissue (J–L), collagen II expression is dramatically reduced, leaving only faint signal within the healing interface (J‐a–L‐a). This pattern supports a faster transition away from cartilage and toward bone formation with Met treatment. Control fractures (M–O) show intense collagen III throughout the callus, indicating a more fibrotic, less remodeled matrix (M‐a–O‐a). In Met‐treated calluses (P–R), collagen III is limited to small residual patches (P‐a–R‐a), demonstrating reduced fibrous tissue and more complete remodeling toward mature bone. Yellow bars: 500 µm; red bars: 200 μm.

Immunofluorescence aligned with histologic observations. Collagen I was minimal within the control fracture gap (Figure 2A–C and A‐a–C‐a) but highly expressed in Met‐treated callus, reflecting robust new bone deposition (Figure 2D–F and D‐a–F‐a). Conversely, collagen II was abundant in control calluses (Figure 2G–I and G‐a–I‐a), confirming persistent cartilage. In Met‐treated rats, collagen II expression was greatly reduced or absent (Figure 2J–L and J‐a–L‐a), demonstrating accelerated cartilage resolution. Collagen III, associated with fibrotic/scar remodeling, was widely distributed in controls (Figure 2M–O and M‐a–O‐a) but limited to small residual traces following Met treatment (Figure 2P–R and P‐a–R‐a), indicating reduced fibrosis and more advanced remodeling.

### 3.4 Qualitative Immunofluorescence Analysis Demonstrates Increased AMPK Signaling in Metformin‐Treated Callus

**Figure 3:**
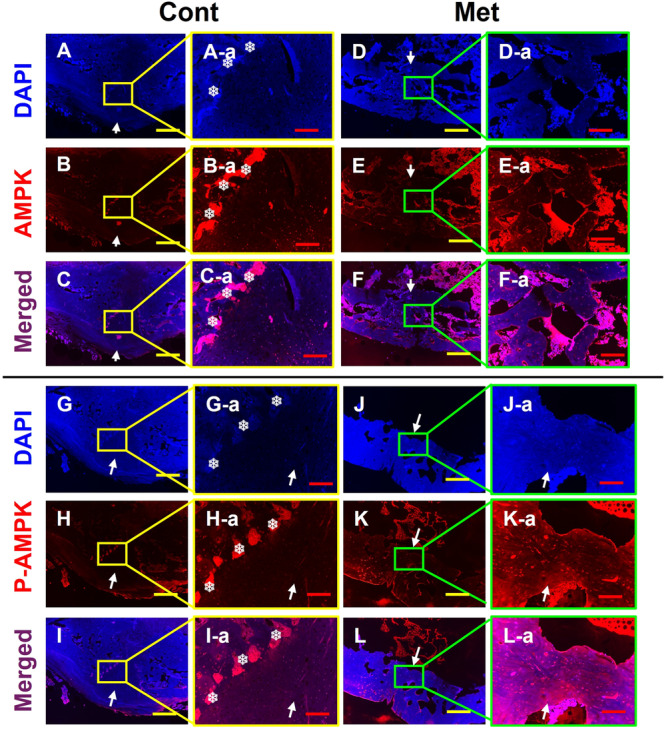
Metformin increases AMPK activation in the fracture callus. AMPK immunofluorescence reveals minimal activation within newly formed tissue in controls (A–C), with expression confined to remaining intact cortical regions (A‐a–C‐a). In striking contrast, Met‐treated calluses (D–F) show intense AMPK activation throughout newly formed woven bone and callus tissue (D‐a–F‐a), indicating metabolic pathway engagement during healing. Consistent with total AMPK staining, p‐AMPK is almost absent in control calluses (G–I), visible only in intact bone (G‐a–I‐a), whereas Met‐treated fractures (J–L) show strong, widespread p‐AMPK signal throughout the repair tissue (J‐a–L‐a). These results confirm that Met activates AMPK signaling within the healing bone microenvironment. Yellow bars: 500 µm; red bars: 200 µm.

AMPK immunofluorescence was substantially higher in Met‐treated calluses (Figure 3D–F and D‐a–F‐a) compared with weak or absent activation in controls (Figure 3A–C and A‐a–C‐a). p‐AMPK showed a parallel expression pattern, with strong phosphorylation in Met‐treated fractures (Figure 3J–L and J‐a–L‐a) and minimal signal in controls (Figure 3G–I and G‐a–I‐a). These findings confirm Met‐dependent AMPK activation during callus maturation.

### 3.5 Qualitative Immunofluorescence Analysis Shows Increased Mitochondrial Markers NDUFB8 and TFAM

**Figure 4:**
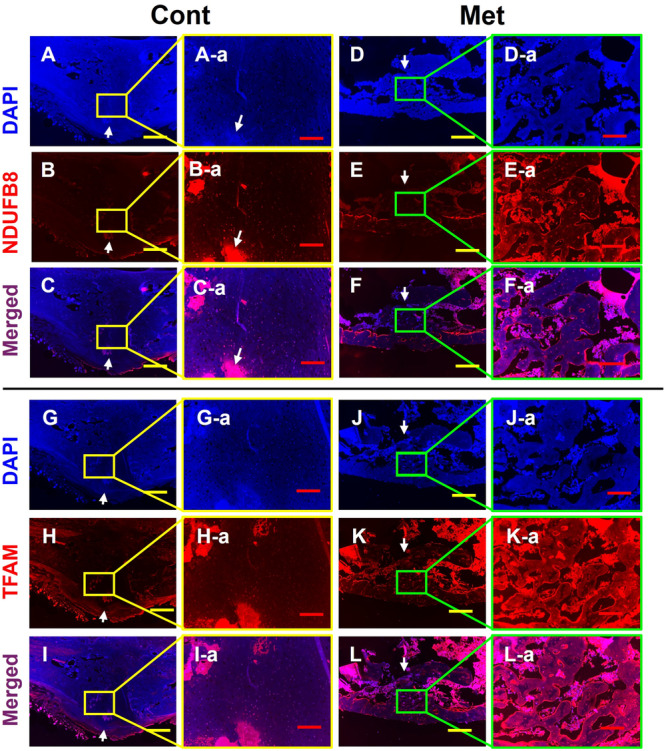
Metformin elevates Mitochondrial Complex I marker NDUFB8 and increases TFAM expression. NDUFB8 expression is weak in control fractures (A–C, A‐a–C‐a), whereas Met‐treated calluses exhibit strong expression across periosteal, intraosseous, and endosteal regions (D–F, D‐a–F‐a). This indicates enhanced mitochondrial metabolic capacity during Met‐stimulated healing. TFAM is sparsely expressed in control tissue (G–I, G‐a–I‐a), but markedly upregulated throughout Met‐treated calluses (J–L, J‐a–L‐a). Together with the strong expression of NDUFB8, this result demonstrates Met‐driven mitochondrial biogenesis during fracture repair. Yellow bars: 500 µm; red bars: 200 µm.

NDUFB8 expression was strongly upregulated throughout the callus in Met‐treated animals (Figure 4D–F and D‐a–F‐a) but weak in controls (Figure 4A–C and A‐a–C‐a). TFAM expression demonstrated a similar trend, with intense periosteal, intraosseous, and endosteal staining in Met‐treated bone (Figure 4J–L and J‐a–L‐a) versus faint, sparse signal in controls (Figure 4G–I and G‐a–I‐a). These data indicate enhanced mitochondrial biogenesis and metabolic capacity under Met treatment.

### 3.6 Qualitative Immunofluorescence Analysis Suggests Reduced HMGB1 Release With Metformin

**Figure 5:**
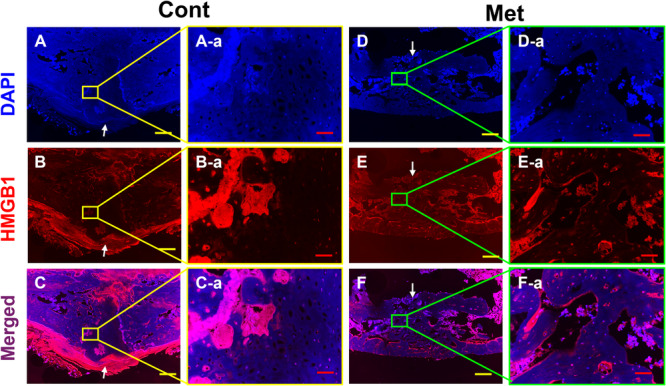
Metformin suppresses HMGB1 release and reduces inflammatory signaling. Control femurs (A–C) show cytoplasmic and extracellular HMGB1 discharge, visible as widespread red/pink staining (B‐a–C‐a), indicative of inflammatory activation. In contrast, Met‐treated calluses (D–F) retain HMGB1 primarily within cell nuclei (blue) and only minimal cytoplasmic staining is observed (D‐a–F‐a), demonstrating reduced inflammatory release of HMGB1 and a more resolved healing environment. Yellow bars: 500 µm; red bars: 100 µm.

HMGB1 staining differed strikingly between groups (Figure 5). Controls showed widespread HMGB1 release into the extracellular space and cytoplasm (Figure 5A–C and A‐a–C‐a). In contrast, Met‐treated calluses retained HMGB1 within nuclei rather than being released extracellularly (Figure 5D–F and D‐a–F‐a), suggesting suppressed inflammatory signaling and reduced cellular stress.

### 3.7 Metformin Accelerates Callus Ossification But Does Not Yet Improve Biomechanics

**Figure 6:**
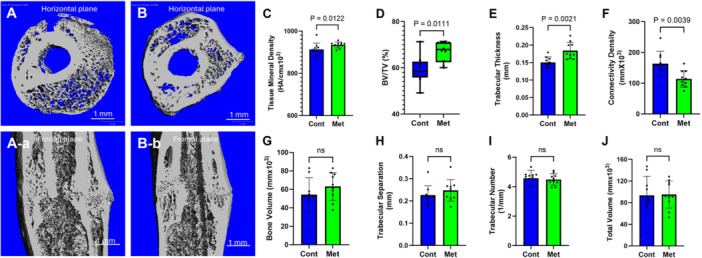
Micro‐CT confirms accelerated callus ossification with metformin. Control calluses (A) show thin, irregular trabeculae with substantial non‐mineralized space, lacking cortical definition (A‐a). Met‐treated fractures (B) exhibit thicker, well‐organized trabeculae and early cortical shell formation (B‐b). Quantification demonstrates significant increases in tissue mineral density, BV/TV, trabecular thickness, with reduced connectivity density (C–F, respectively), whereas bone volume (G) and trabecular separation (H) showed non‐significant favorable trends, however trabecular number and total volume showed no significant (ns) difference (I, J). White bars: 1 mm.

Micro‐CT scans revealed thicker, more uniformly distributed trabeculae with visible cortical shell development in Met‐treated calluses (Figure 6B‐b). Controls exhibited thin, irregular trabeculae and non‐mineralized gaps (Figure 6A‐a). Quantitatively, tissue mineral density, BV/TV (bone volume/total volume), and trabecular thickness (Tb.Th) were significantly higher, and connectivity density significantly lower, in Met‐treated bone (*p* < 0.05, C–F). Other metrics (TV, BV, trabecular number (Tb.N), trabecular separation (Tb.Sp)) trended favorably but were not statistically different (Figure 6G–J).

**Figure 7:**
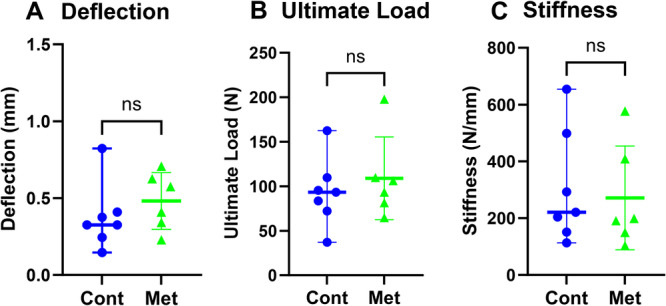
Metformin does not yet alter biomechanical properties at 6 weeks. Quantitative comparison confirms no significant (ns) differences in deflection, ultimate load, or stiffness at this time point (A–C), suggesting that structural maturation precedes mechanical recovery and that this intermediate time point likely precedes full cortical consolidation required for measurable strength improvement. Mechanical changes at this stage are therefore expected to be modest and may fall below the detection sensitivity of the testing method during ongoing fracture remodeling.

Biomechanical testing showed similar load–displacement profiles between groups, with no significant differences in deflection, ultimate load, or stiffness at 6 weeks (Figure 7A–C). As expected, both fracture groups were mechanically inferior to intact femurs, likely reflecting insufficient time for full cortical consolidation. In addition to in vivo experiments, we performed an in vitro study using femur‐derived mouse BMCs. We observed increased Col1 expression in normal and osteogenic media in the Met‐treated group compared to vehicle control at day 12 (Figure S3), and increased OCN expression in both normal and osteogenic media treated with Met compared to control at day 19 (Figure S4).

## 4 Discussion

This study suggests that oral metformin treatment was associated with accelerated fracture callus maturation in a rat open femoral osteotomy model. At 6 weeks post‐injury, metformin‐treated animals exhibited more advanced endochondral ossification, improved collagen matrix organization, and favorable micro‐CT indices, including increased tissue mineral density, bone volume fraction, and trabecular thickness with reduced connectivity density. Although biomechanical properties were not significantly different at this intermediate time point, ultimate load and stiffness trended higher in the metformin group. Collectively, these findings suggest that metformin primarily enhanced structural progression of repair rather than immediate mechanical recovery.

The principal finding of this study was the apparent acceleration of the cartilage‐to‐bone transition during fracture healing. Histological analyses showed reduced residual cartilage, greater woven/spongy bone formation, and more organized collagen fibers in metformin‐treated calluses relative to controls. These observations are consistent with more advanced progression through endochondral ossification, a key phase of secondary fracture repair. Prior studies have reported beneficial skeletal effects of metformin, including promotion of osteogenesis, angiogenesis, and stromal cell function under metabolically stressed conditions [18, 19, 21]. Our findings extend this literature by showing that metformin may also favor structural maturation of fracture callus tissue in traumatic bone healing.

A second notable finding was the increased immunofluorescence signal for AMPK‐related markers together with greater staining intensity of mitochondrial‐associated markers NDUFB8 and TFAM in metformin‐treated calluses. These observations are consistent with enhanced metabolic activity during repair. AMPK is a central regulator of cellular energy balance and has been implicated in osteoblast differentiation, autophagy‐mediated matrix turnover, and coordinated bone remodeling [20, 22]. Because fracture healing is an energetically demanding process requiring active participation of chondrocytes, osteoblasts, and stromal cells, enhanced metabolic signaling may contribute to the accelerated callus maturation observed here. However, these pathway‐related findings were based primarily on immunofluorescence staining, and additional biochemical validation using Western blotting, qPCR, or related approaches will be important in future studies.

The temporal pattern of healing also provides useful insight. Differences between groups were limited at 4 weeks, *most apparent at 6 weeks*, and reduced by 8 weeks, suggesting convergence during later remodeling. This pattern is compatible with the rapid healing kinetics of young rodents, in which cartilage replacement, woven bone deposition, and early remodeling often progress substantially by later time points [12, 28, 29, 30]. Similar time‐dependent benefits have been reported for other biologic or anabolic interventions, including BMP‐2, erythropoietin, and sclerostin inhibition [14, 15, 16]. These findings suggest that metformin may exert its greatest influence during the active reparative/remodeling transition phase rather than at very early or late stages of healing.

From a translational perspective, metformin is an attractive candidate because of its extensive clinical experience, oral availability, and low cost. If similar effects are confirmed in additional preclinical and clinical studies, metformin could represent a practical adjunct strategy for patients at risk for delayed healing or impaired skeletal repair. However, while metformin has a well‐established clinical safety record, its current use is most common in patients with type 2 diabetes, insulin resistance, obesity, or related metabolic disorders. Therefore, generalization to broader fracture populations requires further study. In addition, potential adverse effects—including gastrointestinal intolerance, vitamin B12 deficiency with long‐term use, rare lactic acidosis in predisposed individuals, and occasional liver function abnormalities—should be considered in future clinical translation.

This study also has several strengths, including use of a clinically relevant systemic therapy, multimodal assessment of healing (histology, immunofluorescence, micro‐CT, and biomechanics), and temporal evaluation across multiple healing stages. However, several limitations should be acknowledged. First, the controlled open osteotomy model provides reproducibility but does not fully replicate the heterogeneous fracture morphology of blunt traumatic injury. Second, biomechanical testing was performed only at the 6‐week time point; earlier testing (e.g., 4–5 weeks) may better capture transient functional differences during rapid rodent healing. Third, circulating glucose and insulin levels were not measured, although no overt signs of hypoglycemia, abnormal weight loss, or treatment‐related distress were observed. Moreover, prior studies have shown that metformin does not significantly alter blood glucose levels in normoglycemic (non‐diabetic) subjects [31, 32, 33, 34]. Fourth, histological semi‐quantification was performed in a subset of animals and should be interpreted as supportive in conjunction with the concordant micro‐CT and immunofluorescence findings. Finally, only a single metformin dose was studied, and responses across sex, age, metabolic state, and fixation conditions remain to be determined. Future studies should evaluate dose‐response relationships, earlier and later biomechanical endpoints, molecular confirmation of signaling pathways, and efficacy in aged, diabetic, osteoporotic, or otherwise impaired‐healing models. Testing under more rigid fixation constructs may also help determine whether structural improvements translate into measurable gains in mechanical strength.

In conclusion, oral metformin treatment was associated with accelerated fracture callus maturation, improved matrix organization, and favorable structural indices of healing in a rat open femoral fracture model. These findings support further investigation of metformin as a potential low‐cost adjunct therapy for enhancing skeletal repair and highlight metabolic modulation as a promising strategy in fracture‐healing research.

## Author Contributions

**Vasyl Pastukh:** writing original draft, data curation, investigation, formal analysis, validation. **Jianying Zhang:** methodology, data curation, validation. **Peter G. Alexander:** methodology, data curation. **Satyaj Bhargava:** methodology. **Arshia Shams:** methodology. **Celina Zhao:** methodology. **MaCalus V. Hogan:** writing – review and editing. **James H‐C. Wang:** conceptualization, project administration, methodology, validation, resources, writing – review and editing, supervision.

## Ethics Statement

All animal procedures were approved by the University of Pittsburgh Institutional Animal Care and Use Committee (IACUC# IS00022685).

## Conflicts of Interest

The authors declare no conflicts of interest.

## Supporting information


**Figure S1:** Metformin accelerates hyaline cartilage ossification at 6 weeks post‐surgery and does not affect fracture healing at 4‐ and 8‐week time points. Femoral fracture healing dynamics were assessed in control (A–C) and metformin (D–F) groups at 4‐, 6‐, and 8‐weeks post‐surgery, respectively. At 4 weeks post‐surgery, we observed formation of scar and cartilaginous tissue (A‐a, D‐d, white arrows) in the fracture healing site with superficial ossification of callus (black arrows) in both control and Met groups (A‐a, D‐d). These changes are typical for the early callus remodeling stage. After 6 weeks post‐surgery, in the control group, the fracture healing site was characterized predominantly by cartilaginous tissue formation with some regions of scar tissue (white arrows, B‐b) and increased ossification compared to 4 weeks (B‐b, black arrows), representing an early remodeling stage. Whereas in the Met group, the fracture healing site was composed predominantly of spongy bone (E‐e, black arrows), consistent with a middle remodeling phase. At the 8‐week time point, in both groups we observed mature spongy bone (C‐c, F‐f) and cortical bone formation (C‐c, F‐f, green arrows), indicating a late remodeling stage.

**Figure S2:** Overview images of rat femur in 6 weeks post surgery. Control and Met treated groups in Hematoxylin & Eosin (A, B), Safranin O & Fast Green (C, D), Masson Trichrome (E, F), and Picrosirius Red (G, H). Fracture callus region marked with black boxes in control group (a, c, e, g), and in Met group (b, d, f, h).

**Figure S3:** COL1A1: transition‐phase collagen expression in femur‐derived mouse BMSCs on day 12. Femur‐derived mouse bone marrow stromal/stem cells (BMSCs) were cultured under basal conditions (Norm) or osteogenic differentiation conditions (Osteo) with or without metformin (Met, 150 μg/mL) beginning at plating and replenished at each media change. Cells were fixed at differentiation day 12 and subjected to immunofluorescence staining. A–D: COL1A1 immunofluorescence (red) for Norm (A), Norm + Met (B), Osteo (C), and Osteo + Met (D), imaged using identical acquisition parameters within each staining plate. E–H: DAPI nuclear staining (blue) for the same fields, processed for accurate nuclear segmentation. I–L: Merged images (COL1A1 + DAPI) with segmentation masks illustrating the fixed threshold applied for red‐channel area extraction. M: Quantification of COL1A1‐positive area normalized to DAPI‐positive nuclei counts. Fifteen fields per group were analyzed (five images per well across three independent experiments), and measurements were averaged at the well level prior to group comparison. Red‐channel signal was quantified within a predefined ROI using constant threshold settings within each staining plate and normalized to nuclei counts obtained via object‐based segmentation. Under both normal (Norm) and osteogenic (Osteo) culture conditions, addition of Met significantly increased COL1A1 expression. Scale bar: 100 μm. Statistical analysis was performed using an unpaired two‐tailed *t*‐test.

**Figure S4:** OCN: late osteogenic maturation marker expression in femur‐derived mouse BMSCs on day 19. Femur‐derived mouse bone marrow stromal/stem cells (BMSCs) were cultured under basal conditions (Norm) or osteogenic differentiation conditions (Osteo) with or without metformin (Met, 150 μg/mL) beginning at plating and replenished at each media change. Cells were fixed at differentiation day 19 and subjected to immunofluorescence staining. A–D: OCN immunofluorescence (red) for Norm (A), Norm + Met (B), Osteo (C), and Osteo + Met (D), imaged using identical acquisition parameters within each staining plate. E–H: DAPI nuclear staining (blue) for the same fields, processed for accurate nuclear segmentation. I–L: Merged images (OCN + DAPI) with segmentation masks illustrating the fixed threshold applied for red‐channel area extraction. M: Quantification of OCN‐positive area normalized to DAPI‐positive nuclei counts. Fifteen fields per group were analyzed (five images per well across three independent experiments), and measurements were averaged at the well level prior to group comparison. Red‐channel signal was quantified within a predefined ROI using constant threshold settings within each staining plate and normalized to nuclei counts obtained via object‐based segmentation. Under both normal (Norm) and osteogenic (Osteo) culture conditions, addition of Met significantly increased OCN expression. Scale bar: 100 μm. Statistical analysis was performed using an unpaired two‐tailed *t*‐test.

## Data Availability

The data that support the findings of this study are available from the corresponding author upon reasonable request.
