# Supplementary figures and images for: Repurposing Metformin to Promote Fracture Callus Maturation via AMPK‐Driven Metabolic Activation
Source: J Orthop Res. 2026 Jul 4;44(7):e70246. doi: 10.1002/jor.70246 (PMC13332414; doi:10.1002/jor.70246)

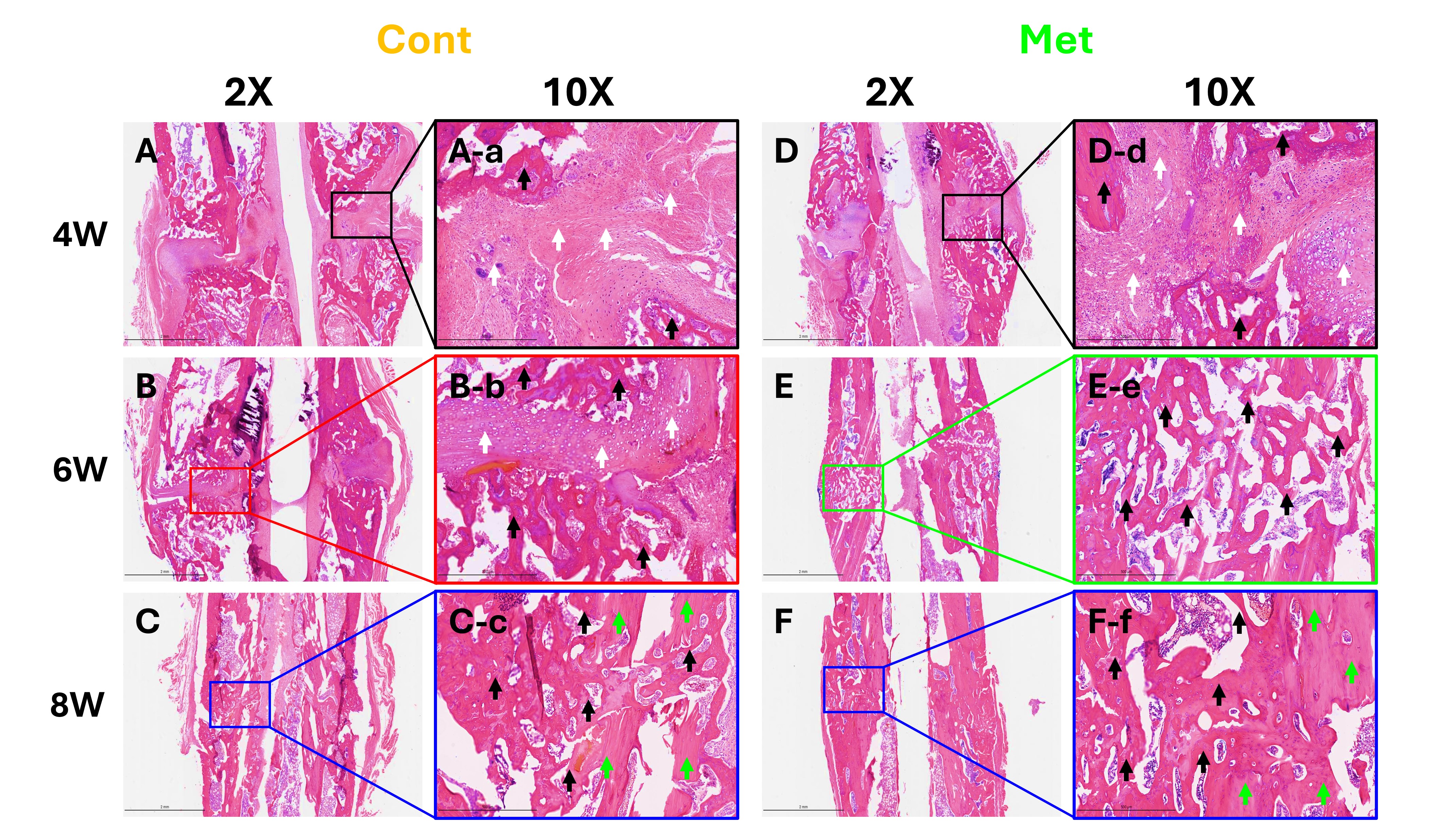

Supplement: Supplementary file 1 — Figure S1: Metformin accelerates hyaline cartilage ossification at 6 weeks post‐surgery and does not affect fracture healing at 4‐ and 8‐week time points. Femoral fracture healing dynamics were assessed in control (A–C) and metformin (D–F) groups at 4‐, 6‐, and 8‐weeks post‐surgery, respectively. At 4 weeks post‐surgery, we observed formation of scar and cartilaginous tissue (A‐a, D‐d, white arrows) in the fracture healing site with superficial ossification of callus (black arrows) in both control and Met groups (A‐a, D‐d). These changes are typical for the early callus remodeling stage. After 6 weeks post‐surgery, in the control group, the fracture healing site was characterized predominantly by cartilaginous tissue formation with some regions of scar tissue (white arrows, B‐b) and increased ossification compared to 4 weeks (B‐b, black arrows), representing an early remodeling stage. Whereas in the Met group, the fracture healing site was composed predominantly of spongy bone (E‐e, black arrows), consistent with a middle remodeling phase. At the 8‐week time point, in both groups we observed mature spongy bone (C‐c, F‐f) and cortical bone formation (C‐c, F‐f, green arrows), indicating a late remodeling stage. [file JOR-44-0-s002.jpg]

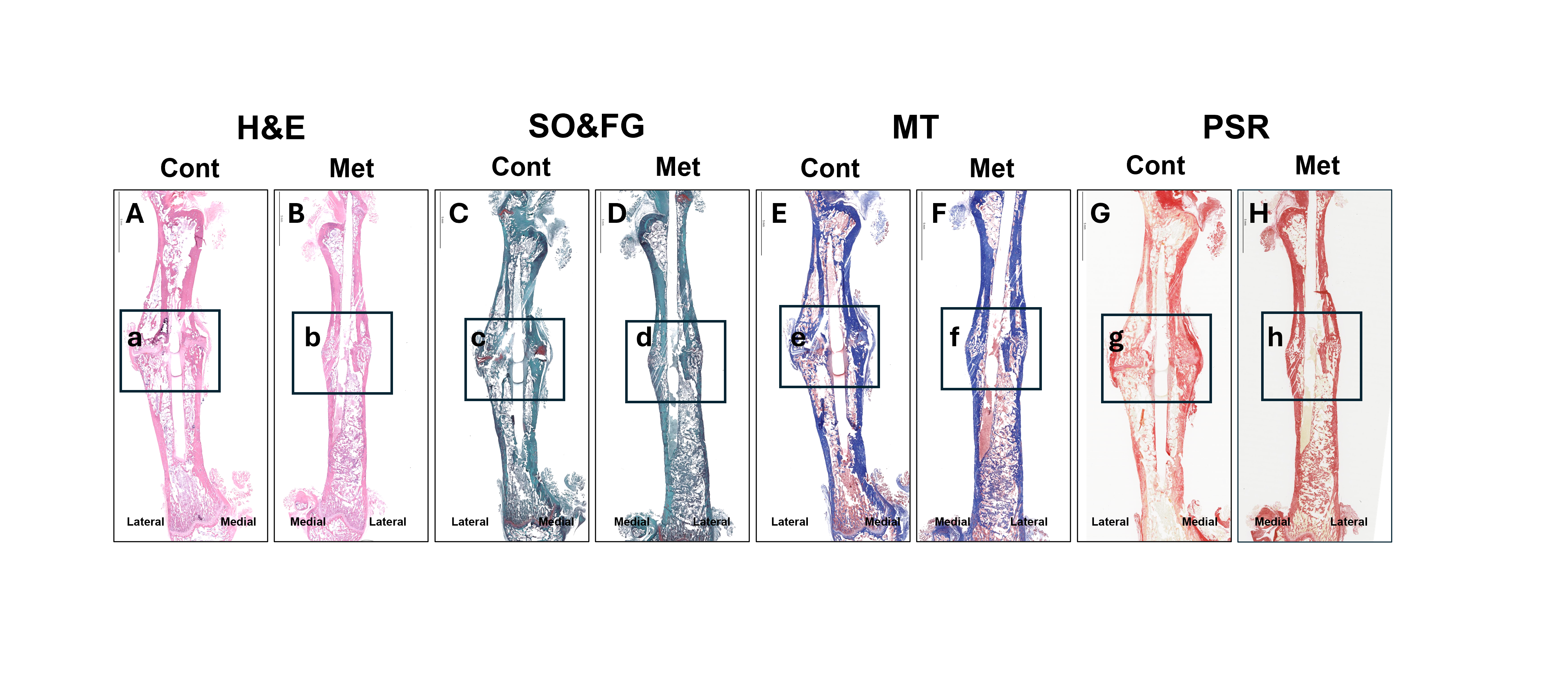

Supplement: Supplementary file 2 — Figure S2: Overview images of rat femur in 6 weeks post surgery. Control and Met treated groups in Hematoxylin & Eosin (A, B), Safranin O & Fast Green (C, D), Masson Trichrome (E, F), and Picrosirius Red (G, H). Fracture callus region marked with black boxes in control group (a, c, e, g), and in Met group (b, d, f, h). [file JOR-44-0-s004.tif]

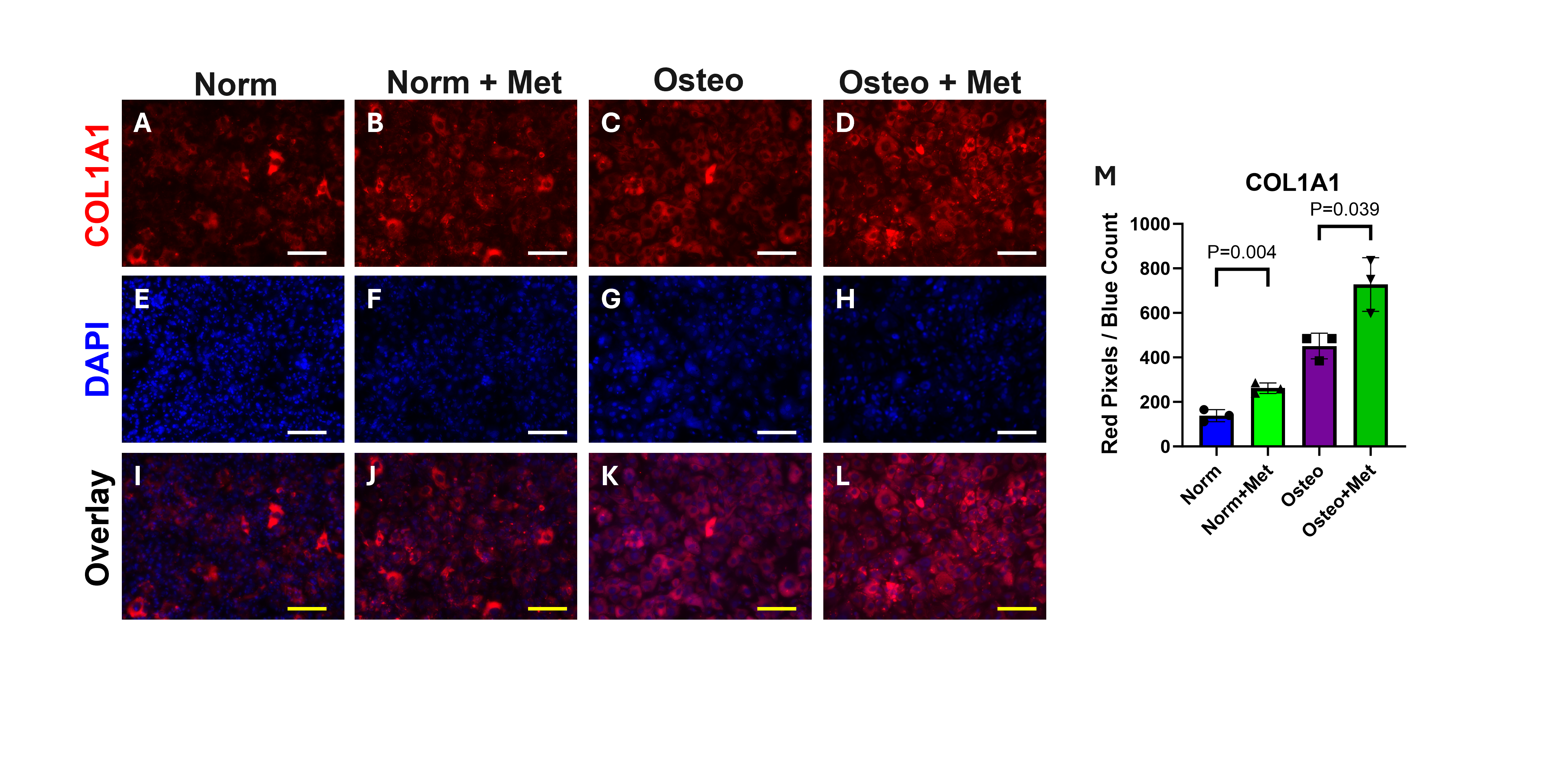

Supplement: Supplementary file 3 — Figure S3: COL1A1: transition‐phase collagen expression in femur‐derived mouse BMSCs on day 12. Femur‐derived mouse bone marrow stromal/stem cells (BMSCs) were cultured under basal conditions (Norm) or osteogenic differentiation conditions (Osteo) with or without metformin (Met, 150 μg/mL) beginning at plating and replenished at each media change. Cells were fixed at differentiation day 12 and subjected to immunofluorescence staining. A–D: COL1A1 immunofluorescence (red) for Norm (A), Norm + Met (B), Osteo (C), and Osteo + Met (D), imaged using identical acquisition parameters within each staining plate. E–H: DAPI nuclear staining (blue) for the same fields, processed for accurate nuclear segmentation. I–L: Merged images (COL1A1 + DAPI) with segmentation masks illustrating the fixed threshold applied for red‐channel area extraction. M: Quantification of COL1A1‐positive area normalized to DAPI‐positive nuclei counts. Fifteen fields per group were analyzed (five images per well across three independent experiments), and measurements were averaged at the well level prior to group comparison. Red‐channel signal was quantified within a predefined ROI using constant threshold settings within each staining plate and normalized to nuclei counts obtained via object‐based segmentation. Under both normal (Norm) and osteogenic (Osteo) culture conditions, addition of Met significantly increased COL1A1 expression. Scale bar: 100 μm. Statistical analysis was performed using an unpaired two‐tailed t‐test. [file JOR-44-0-s001.tif]

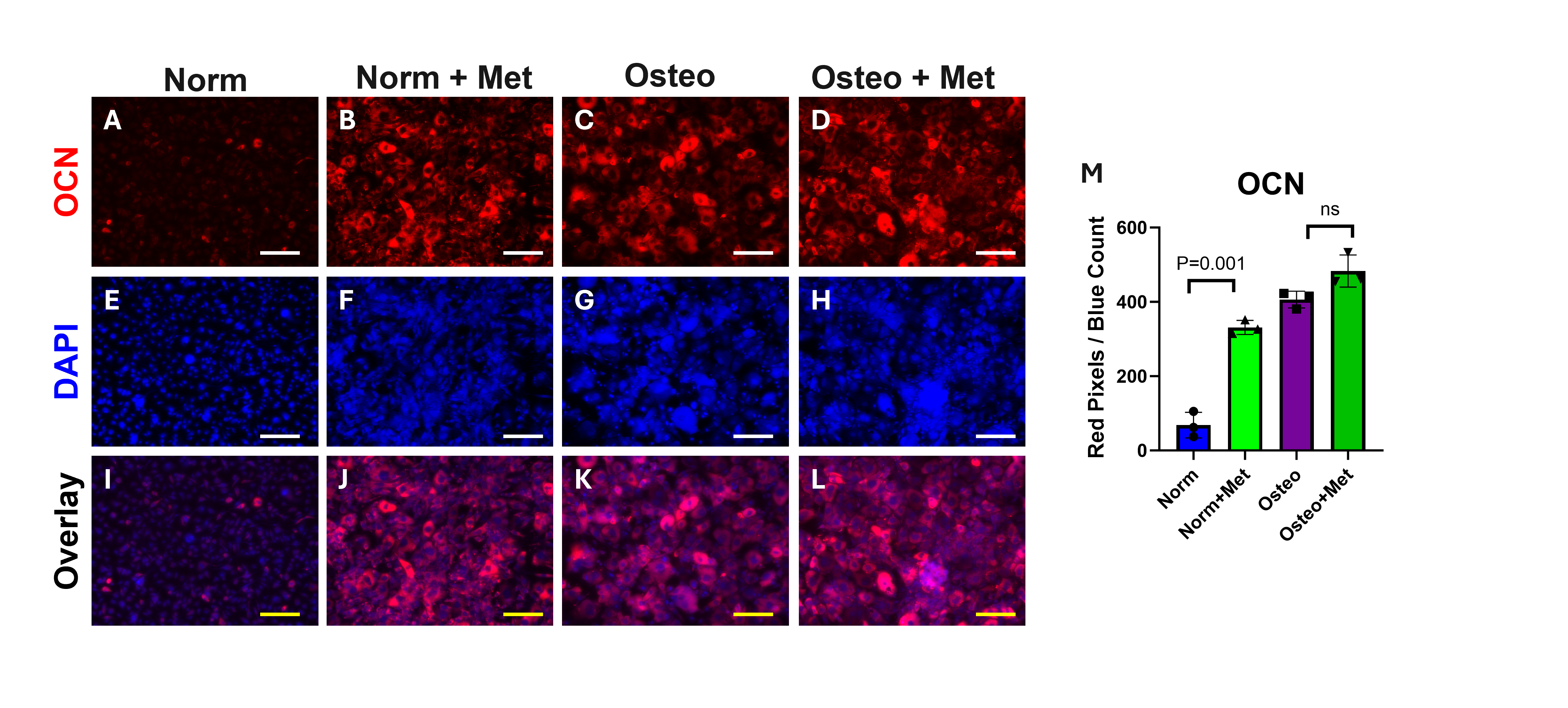

Supplement: Supplementary file 4 — Figure S4: OCN: late osteogenic maturation marker expression in femur‐derived mouse BMSCs on day 19. Femur‐derived mouse bone marrow stromal/stem cells (BMSCs) were cultured under basal conditions (Norm) or osteogenic differentiation conditions (Osteo) with or without metformin (Met, 150 μg/mL) beginning at plating and replenished at each media change. Cells were fixed at differentiation day 19 and subjected to immunofluorescence staining. A–D: OCN immunofluorescence (red) for Norm (A), Norm + Met (B), Osteo (C), and Osteo + Met (D), imaged using identical acquisition parameters within each staining plate. E–H: DAPI nuclear staining (blue) for the same fields, processed for accurate nuclear segmentation. I–L: Merged images (OCN + DAPI) with segmentation masks illustrating the fixed threshold applied for red‐channel area extraction. M: Quantification of OCN‐positive area normalized to DAPI‐positive nuclei counts. Fifteen fields per group were analyzed (five images per well across three independent experiments), and measurements were averaged at the well level prior to group comparison. Red‐channel signal was quantified within a predefined ROI using constant threshold settings within each staining plate and normalized to nuclei counts obtained via object‐based segmentation. Under both normal (Norm) and osteogenic (Osteo) culture conditions, addition of Met significantly increased OCN expression. Scale bar: 100 μm. Statistical analysis was performed using an unpaired two‐tailed t‐test. [file JOR-44-0-s003.tif]
